# Synthesis of Ganoderic Acids Loaded Zein-Chitosan Nanoparticles and Evaluation of Their Hepatoprotective Effect on Mice Given Excessive Alcohol

**DOI:** 10.3390/foods13172760

**Published:** 2024-08-29

**Authors:** Yingjia Cao, Yuheng Yang, Zihua Liang, Weiling Guo, Xucong Lv, Li Ni, Youting Chen

**Affiliations:** 1 Department of Hepatopancreatobiliary Surgery, Fujian Research Institute of Abdominal Surgery, The First Affiliated Hospital of Fujian Medical University, Fuzhou 350005, China; 220820074@fzu.edu.cn (Y.C.); yangyh6191003072@fjmu.edu.cn (Y.Y.); 2 Institute of Food Science and Technology, College of Biological Science and Technology, Fuzhou University, Fuzhou 350108, China; 220820089@fzu.edu.cn (Z.L.); weilguo@fzu.edu.cn (W.G.); nili@fzu.edu.cn (L.N.); 3 Food Nutrition and Health Research Center, School of Advanced Manufacturing, Fuzhou University, Jinjiang 362200, China; 4 The First Affiliated Hospital of Fujian Medical University, Fujian Medical University, Fuzhou 350004, China; 5 Department of Hepatopancreatobiliary Surgery, National Regional Medical Center, Binhai Campus of the First Affiliated Hospital, Fujian Medical University, Fuzhou 350212, China

**Keywords:** ganoderic acids, zein-chitosan nanoparticles, alcoholic liver injury, hepatoprotective effects, intestinal microbiota, mRNA expression

## Abstract

*Ganoderma lucidum*, used in East Asia for its health benefits, contains ganoderic acids (GA) which have various pharmacological activities but are limited by poor water solubility and low oral bioaccessibility. This study synthesized and characterized ganoderic acids loaded zein-chitosan nanoparticles (GA-NPs), and investigated its advantages in alleviating alcoholic liver injury (ALI) in mice model. The GA-NPs demonstrated high encapsulation efficiency (92.68%), small particle size (177.20 nm), and a +29.53 mV zeta potential. The experimental results of alcohol-induced liver injury mouse model showed that GA-NPs significantly improved liver metabolic function, reduced alcohol-induced liver oxidative stress in liver by decreasing lactate dehydrogenase activity and malondialdehyde level, while increasing the activities of liver antioxidant enzymes and alcohol dehydrogenase. Moreover, GA-NPs were favorable to ameliorate intestinal microbiota dysbiosis in mice exposed to alcohol by increasing the proportion of probiotics such as *Romboutsia*, *Faecalibaculum*, *Bifidobacterium* and *Turicibacter*, etc., which were highly correlated with the improvement of liver function. Furthermore, GA-NPs modulated the mRNA expression related to ethanol metabolism, oxidative stress and lipid metabolism. Conclusively, this study revealed that GA-NPs have stronger hepatoprotective effects than non-encapsulated ganoderic acids on alleviating ALI by regulating intestinal microbiota and liver metabolism.

## 1. Introduction

As one of the most beloved drinks in the world, alcoholic beverages, especially the high-alcohol wines, are consumed by billions of people around the world for centuries. In the last decade, the growing health problems caused by chronic or heavy alcohol consumption have become a major public health issue worldwide. Alcoholic liver disease (ALD) is a disease caused by excessive alcohol intake and characterized by liver tissue lesions, which may be further induced into fatty liver, hepatitis, cirrhosis and even liver cancer [1]. With high morbidity and mortality, ALD results in a huge economic burden to people. Excessive alcohol intake has been reported to cause abnormalities in the composition of gut bacteria, destroy the intestinal barrier function and intestinal mucosal integrity [2]. In particular, the damage to the intestinal barrier and the increase of gram-negative bacteria can result in the massive transfer of gut-derived endotoxin into the portal bloodstream, delivering a “second blow” to the liver, and thus exacerbating the pathological process of liver disease [3]. It was reported that alcohol abuse significantly increases the abundance of *Enterococcus* and *Klebsiella* in the gut, which are strongly associated with the severity of liver disease and mortality in ALD patients [4]. To date, the pathogenesis of ALD has not been fully clarified, which may be related to the toxicity of ethanol metabolite acetaldehyde, intestinal-derived endotoxins, cytokines, oxidative stress and lipid oxidation of hepatocytes [1]. During the metabolism of ethanol in the liver, toxic metabolites such as acetaldehyde and reactive oxygen free radicals are produced, causing oxidative stress, lipid peroxidation, endoplasmic reticulum stress and other reactions in hepatocytes, potentially leading to liver injury. Drug therapy is the primary treatment for patients with ALD, but there are no completely effective drugs for the treatment of ALD [5,6]. Besides, most of the drugs (e.g., naltrexone) used to treat alcohol dependence tend to cause withdrawal symptoms when taken, and the treatment of acamprosate was failed to confirm in USA [7]. Considering this worrying scenario, it is urgent to discover bioactive ingredients with a strong hepatoprotective effect from natural food resources to prevent or delay the pathological development of ALD.

*Ganoderma lucidum*, also known as “*Reishi*”, is a large fungus popular in East Asia for both medicinal and dietary purposes, due to its diverse biological activities and attractive pharmacological characteristics in various human diseases, making it a promising market with broad development prospects. It has been utilized as a conventional medicine and food in China with a long history. There is growing evidence that the fruit body of *G. lucidum* is rich in a variety of health-promoting components, including polysaccharides, triterpenoids and polypeptides. Ganoderic acids (GA) are the most commonly discovered triterpenoids in *G. lucidum* fruiting body and exhibit diverse pharmacological activities, including anti-tumor, immunomodulator, anti-inflammation, hepatoprotective, hypocholesterolemic, hypoglycaemic, hypolipidemic and cardioprotective properties [8,9,10]. In addition, GA has promising potential in preventing liver damage caused by high-fat diets and excessive alcohol exposure [11,12]. However, the poor water solubility and low oral bioaccessibility of GA greatly limit its application in commercial health foods [13]. Therefore, designing an efficient delivery system to encapsulate GA is considered to be a potentially effective solution to enhance the hepatoprotective activity and improve the therapeutic efficacy of GA [14].

The encapsulation of bioactive components in a biopolymer matrix has been widely studied to enhance the stability and bioaccessibility of these components in the digestive tract, allowing them to exert their beneficial effects. Among biopolymer-based encapsulation, nano-encapsulation technology is widely accepted for its outstanding advantages, which is defined as embedding bioactive compounds in nano-scale carriers [15]. Food-derived proteins and polysaccharides and their nanocomplexes, are promising feedstocks for development due to their high safety, low cost and good bioavailability. As a typical hydrophobic protein, zein is widely accepted in the encapsulation of hydrophobic compounds, due to its biocompatibility, biodegradability and self-assembly ability [16]. However, the use of zein particles as a nano-delivery vehicle is still limited by its poor solubility in water and acidic conditions. When exposed to a certain pH, salt or high temperature environment, it would make the whole nano-delivery system unstable and unfavourable for the embedded delivery of certain bioactive substances [17]. One of the feasible strategies to overcome these limitations is to form nanocomplexes of zein with polysaccharides to enhance the stability of zein. As the only natural alkaline polysaccharide discovered to date, chitosan is widely used as a nanoparticle carrier for encapsulating bioactive compounds due to its ability to prevent digestive enzyme degradation and control release [18]. Chitosan has a special ability to adhere to the mucosal surface of the gastrointestinal tract, which is conducive to prolonging the retention time of bioactive compounds in the body and improving their bioavailability [19]. In recent years, chitosan has been commonly used to create a structural layer around zein nanoparticles to prepare zein-chitosan nanocomposites with a unique structure, which improves aggregation stability by increasing steric resistance and electrostatic repulsion. Moreover, chitosan improves the capture efficiency and encapsulation rate of bioactive molecules. Previous research has shown that zein/chitosan-based nanoparticles can be used to load various bioactive ingredients (such as curcumin, alpha-tocopherol, and oral DNA), and have been shown to control release in in vitro and in vivo experiments [20]. To date, the potential of zein/chitosan-based nanoparticles to improve the hepatoprotective effect of GA has not been explored.

In this study, we synthesized and characterized ganoderic acids loaded zein-chitosan nanoparticles (GA-NPs), and investigated its advantages on alleviating ALI in mice exposed to alcohol. The hepatoprotective effects and mechanisms of GA-NPs were elucidated by biochemistry, histopathology and gene transcription analysis. High-throughput sequencing technology was used to study the composition of intestinal microbiome. The possible relationships between intestinal bacteria and biochemical phenotypes were revealed by correlation network analysis, in order to reveal the enhancement of the liver-protecting efficacy of GA encapsulated by zein-chitosan nanoparticles and its associated mechanisms of action.

## 2. Materials and Methods

### 2.1. Materials and Reagents

*G. lucidum* fruiting body used for the preparation of GA was obtained from Zhejiang Keda Biotech. Co., Ltd. (Lishui, Zhejiang, China). Zein (>92% purity) and chitosan (CS, degree of deacetylation of 85%) were supplied by Sigma-Aldrich^®^ Inc. (St. Louis, MO, USA). The antioxidant and biochemical assay kits for total cholesterol (TC), triglyceride (TG), high-density lipoprotein cholesterol (HDL-C), low-density lipoprotein cholesterol (LDL-C), glutathione (GSH), superoxide dismutase (SOD), catalase (CAT), malondialdehyde (MDA) and lactate dehydrogenase (LDH) were sourced from Elabscience^®^ Biotech. Co., Ltd. (Wuhan, Hubei, China). Unless otherwise noted, this study utilized analysis-grade chemicals acquired from Xilong Scientific Co., Ltd. (Foshan, Guangdong, China).

### 2.2. Synthesization of GA-NPs

GA were extracted and purified from the fruiting body of *G. lucidum* according to our previously published work [12]. GA-NPs were synthesized through anti-solvent precipitation method as described in a prior research [20] with minor modifications. To be more precise, zein (10 mg) was weighed and dissolved in 80% (*v*/*v*) ethanol-water solution (10 mL) and stirred at 1000 rpm for 5 min to create a zein reserve solution with a mass concentration of 1 mg/mL. GA was added to a certain amount of zein solution and stirred continuously at 1000 rpm for 5 min to promote dissolution. GA-NPs were obtained by dissolving a certain amount of CS solution (1% *w*/*v* acetic acid) in a certain amount of GA-zein mixture after 30 min of continuous stirring (1000 rpm). In detail, the concentration of CS was controlled at 0.2, 0.6, 1.0, 1.4 and 1.8 mg/mL. The ethanol in the suspension is evaporated through a rotary evaporator (80 rpm, 50 °C for 8 min), and then centrifuged at 3000× *g* for 5 min to remove the unencapsulated GA. Finally, the prepared GA-NPs were used for particle characterization or frozen dried for animal experiments.

### 2.3. Nanoparticle Characteristics

#### 2.3.1. Particle Size, Polydispersity Index (PDI) and ζ-Potential Measurement

The measurement of particle size, PDI and ζ-potential were executed using Nanosizer ZS-90 (Malvern Instruments Ltd., Worcestershire, UK) according to a prior described method [21]. Before analysis, the samples were diluted with distilled water to get a measurable signal and prevent multiple scattering events. All measurments were repeated in triplicate.

#### 2.3.2. Encapsulation Efficiency and Loading Efficiency Determination

The amount of GA encapsulated in the GA-NPs was measured by a modified colorimetric method [22]. A certain amount of nanoparticles were centrifuged at 1500× *g* for 5 min after a series of treatments. GA content in the supernatant was determined by UV-visible spectroscopy (Lambda 35, PerkinElmer, Waltham, MA, USA) at a wavelength of 545 nm. The concentration of GA was measured by using a standard curve established ranging from 0.2 to 1.0 mg/mL of standard solution (R^2^ = 0.998). 

#### 2.3.3. Scanning Electron Microscopy (SEM) Analysis

The morphology of the nanoparticles was observed by using SEM (Nova NanoSEM 230, Brno, Czech Republic). Samples were placed on an electric conductor and then sprayed twice with a thin layer of gold under high vacuum conditions. The acceleration voltage for testing was 6 kV. 

#### 2.3.4. Fourier Transform Infrared (FT-IR) Spectroscopy

The chemical structures of freeze-dried samples were obtained by FT-IR spectrometer (AVATAR360, Thermo Fisher Scientific, Waltham, MA, USA). The scanning ranged from 400 cm^−1^ to 4000 cm^−1^ with a resolution of 4 cm^−1^. The samples were pressed into thin sheets with KBr prior to spectrum capture.

### 2.4. Animal Treatment and Sample Collection

Forty-eight Kunming mice (6 weeks old, male, specific pathogen-free) were obtained from Beijing HFK Bioscience Co., Ltd. (Beijing, China) and raised under the standard environment (22 ± 1 °C, 50–55% humidity, 12 h light/dark cycle) with ad libitum water and common feedstuff. After a seven-day acclimatization, the mice were randomly assigned to the following six groups: (1) the control group (n = 8); (2) the model group (n = 8); (3) the GA-L group (n = 8, GA dosage: 12 mg/kg b.w./day); (4) the GA-H group (n = 8, GA dosage: 36 mg/kg b.w./day); (5) the GA-NPs-L group (n = 8, GA dosage: 12 mg/kg b.w./day); (6) the GA-NPs-H group (n = 8, GA dosage: 36 mg/kg b.w./day). Mice in the model, GA-L, GA-H, GA-NPs-L and GA-NPs-H groups were orally gavaged with 50% alcohol solution (*v*/*v*, 7.5 mL/kg b.w./day), either alone or in combination with administration of GA or GA-NPs suspension once a day. In the control group, the mice were offered an equal volume of physiological saline. We recorded the body weight of each mouse weekly throughout the duration of the experiment. After a six-week intervention period, all mice underwent overnight fasting and subsequently euthanized under anesthesia. Fecal samples were collected 1 days before the end of the experiment and collected after fasting for the determination of SCFAs. Blood samples were allowed to stand at room temperature for 4 h and then centrifuged (3000 rpm) for at 25 °C for 10 min. The obtained serum was kept in sterile centrifuge tubes at −20 °C for biochemical detection. Upon dissection, the liver, spleen, kidney and colon of each mouse were weighed. The liver and cecal contents were quickly frozen in liquid nitrogen and then stored at −80 °C for subsequent analysis.

### 2.5. Biochemical Assays of the Serum and Liver Samples

The levels of LDL-C, HDL-C, TC, TG, aspartate transaminase (AST) and alanine aminotransferase (ALT) in serum were detected by the automatic biochemical analyzer (Toshiba TBA-40FR, Tokyo, Japan). Based on the instructions provided by the kit manufacturer, a homogenizer was utilized to prepare 10% liver tissue homogenate for the analysis of liver biochemical indicators. The supernatant was collected after high-speed centrifugation (10,000× *g* for 10 min, 4 °C). The hepatic levels of GSH, CAT, MDA, SOD, TC, TG, LDH and ADH were measured by the corresponding commercial kits.

### 2.6. Histopathologic Evaluation of Liver and Jejunum Tissues

To evaluate the histopathological situation, both fresh liver and jejunum sections of each mouse were fixed with 4% paraformaldehyde at room temperature for 24 h. Subsequently, the treated liver and jejunum sections were subjected to a series of dehydrated steps using ethanol and xylene solutions, followed by embedding in paraffin. The embedded samples were then sliced into 5 μm thick sections. After preparation, the liver and jejunum sections were stained with hematoxylin and eosin (H&E), and then observed with a light microscope and captured with a digital camera (Nikon, Tokyo, Japan). 

### 2.7. Determination of Short-Chain Fatty Acids (SCFAs) in Fecal Samples

SCFAs concentrations were measured as a previously described method [11]. Specifically, dry feces (100 mg) were suspended in 1.0 mL distilled water, swirled for 2 min, then acidified with 600 μL of 20% (*v*/*v*) H_2_SO_4_, swirled for 1 min, added 500 μL n-butanol, swirled for 2 min, mixed evenly and centrifuged (repeated 3 times). After the supernatant was filtrated by 0.22 μm filter membrane, the SCFAs components in feces were detected by Agilent 7890B gas chromatograph (GC) (Agilent, Santa Clara, CA, USA). A DB-FFAP capillary column (30 m × 0.32 mm id, 0.25 µm film thickness) was employed for GC detection (Agilent, Santa Clara, CA, USA).

### 2.8. High Throughput Sequencing of 16S rDNA Amplicon

Bacterial genomic DNA in cecum content samples was extracted using a fecal bacterial total DNA extraction kit (MoBio, San Mateo, CA, USA). The V3-V4 hypervariable region of bacterial 16S rDNA was amplified using 341F-806R universal primers, and sequenced on the Illumina NovaSeq 6000 platform at Majorbio Co., Ltd. (Shanghai, China) in accordance with our prior research protocol [11]. QIIME 2.0 software (ver. 2019.7) was used for quality control and cluster analysis of the sequencing data, and the refined sequencing data (filtered sequences) were matched against the sequences within the GreenGenes database (Ver. 13.8) to characterize the bacterial phylotypes present in the intestines at the genus level (with more than 97% similarity). Intestinal flora composition was analyzed using principal component analysis (PCA) and hierarchical cluster analysis in SIMCA software (ver. 14.1). Genus-level variations between the different experimental groups were analyzed and visualized through STAMP software (ver. 2.1.3) based on the Welch t-test. Spearman’s correlation analysis was employed to examine the association between intestinal bacteria and biochemical parameters, and visualized through R program (ver. 4.1.2) and Cytoscape (ver. 3.10.1). 

### 2.9. Reverse Transcription-Quantitative Polymerase Chain Reaction (RT-qPCR)

RNA extraction from mouse liver was performed using a miRNeasy mini kit (Vazyme, Nanjing, China), and the extracted RNA was determined by a nucleic acid quantifier (Implen, Munich, Germany). Then the mRNA was reverse-transcribed into cDNA using a commercial kit (Takara, Beijing, China) containing gDNA scavenger. RT-qPCR was performed in GeneLight 9820 quantitative PCR system (Anpuli Biological Engineering Co., Ltd., Xiamen, China) using SYBR^®^ FAST qPCR Master Mix (Vazyme, Nanjing, China) with primers targeting specific genes. The transcription levels of all target genes were normalized using 18S rRNA gene. The RT-qPCR data were assessed utilizing the standard 2^−ΔΔCT^ method for analysis. The PCR primers used in this study are shown in Table 1.

### 2.10. Statistical Analysis

The experimental results were expressed as the mean ± standard deviations. Student’s t-test was performed using GraphPad Prism software (ver. 9.0) and differences were considered statistically significant at *p* < 0.05.

## 3. Results and Discussion

### 3.1. Effects of CS Concentration on GA-NPs Characteristics

**Figure 1:**
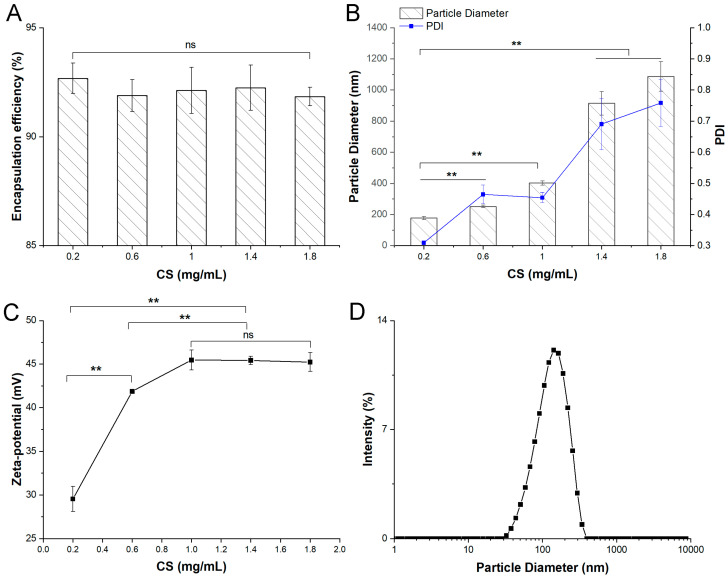
Characterization of zein-chitosan nanoparticles loaded with ganoderic acids (GA-NPs). (**A**) Encapsulation efficiency of ganoderic acids in GA-NPs; (**B**) Particle diameter and polydispersity index (PDI) of GA-NPs; (**C**) ζ-potential of GA-NPs; (**D**) Particle diameter distribution of GA-NPs (** *p* < 0.01).

The effects of CS concentrations on particle size and PDI of GA-NPs are illustrated in Figure 1. With the increase of CS concentration from 0.2 mg/mL to 1.6 mg/mL, the particle size increased sharply from 177.20 nm to 1086.43 nm. The interaction between hydrogen bonds increased with the increase of CS concentration, and then forming a larger self-assembled nano-polymer, resulting in a gradual increase in the particle size of the composite. After mixing CS, GA and zein in solution, nanoparticles spontaneously formed with a positive surface charge, measured by zeta potential (29.53–45.23 mV) (*p* < 0.01, versus GA). When CS concentration reached 0.2 mg/mL, the PDI of nanoparticles was the smallest (0.3097), indicating that CS concentration has a great influence on the PDI of nanoparticles (*p* < 0.01, versus GA). The encapsulation efficiency was defined by the percentages of GA in zein/CS nanoparticles. When the amount of zein and GA remained constant, there was no significant difference in encapsulation efficiency with the increase of CS concentration, and the encapsulation efficiencies of all nanoparticles were above 90%. Therefore, under the optimal CS concentration (0.2 mg/mL), the prepared GA-NPs had an average diameter of 177.20 ± 10.45 nm, an encapsulation efficiency of 92.68%, a PDI of nanoparticles of 0.3197 ± 0.0065, and the zeta potential of 29.53 ± 1.44 mV. These results indicate that the prepared GA-NPs has the advantages of small particle size and uniform particle size distribution. 

### 3.2. Morphological Observation of GA-NPs

**Figure 2:**
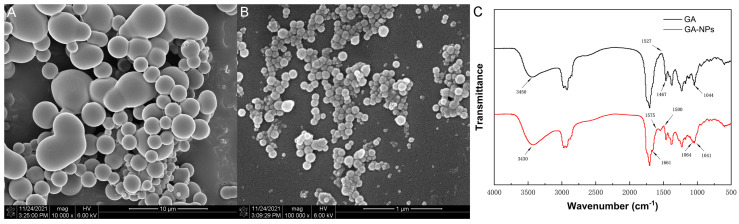
Microstructure characterization of GA-NPs by SEM and FT-IR spectroscopy. (**A**) SEM image of GA; (**B**) SEM image of GA-NPs; (**C**) FT-IR spectra of GA and GA-NPs. Note: The chitosan (CS) concentration in GA-NPs for microstructure characterization was 0.02 mg/mL.

The micromorphology of GA-NPs was evaluated by scanning electron microscopy. The synthesized nanoparticles exhibited an approximate spherical structure with uniform size and smooth surface, and the size of nanoparticles was 100–200 nm (Figure 2A). The particle size results of GA-NPs under SEM are similar to the results of dynamic light scattering (DLS), both within the range of 100–200 nm. The above results indicate that GA-NPs have been successfully prepared by the self-assembly of GA with zein and chitosan.

### 3.3. FT-IR Characterization of GA-NPs

In order to further characterize the interaction pathway of chemical bonds between GA, CS and zein, FT-IR spectroscopy was performed for GA and GA-NPs. The inter-molecular interactions of GA and GA-NPs were investigated by measuring the shift of specific peaks in the FT-IR spectra. As shown in the Figure 2B,C, GA exhibited marked O-H stretching at 3450 cm^−1^, and -CH2-bending vibration and C-O stretching vibration existed at 1467 cm^−1^ and 1044 cm^−1^, respectively [23]. In the spectrum of GA-NPs, the hydrogen bond band was 3430 cm^−1^, which is consistent with the strong hydrogen bond between zein and chitosan. The stretching vibration peak of hydrogen bond in GA-NPs migrated from 3450 cm^−1^ to 3430 cm^−1^, which may be due to the interaction of the carbonyl group in the amide bond on zein with the hydroxyl group on the CS molecule [24]. In addition, compared with GA, GA-NPs performed a new peak at 1661 cm^−1^, representing the vibration of the amide I (C=O) group. Meanwhile, GA-NPs showed multiple peaks at 1575 cm^−1^, 1500 cm^−1^ and 1064 cm^−1^, corresponding to amide II (N-H bending), carboxyl groups and C-O-C stretching, indicating that the hydrophobic interaction and electrostatic interaction in GA-NPs were enhanced. Moreover, zein contains highly hydrophobic molecules, so hydrophobic interactions may also contribute to the formation of nanoparticles. 

### 3.4. Effects of GA-NPs Intervention on the Body Weight and Organ Indexes

**Figure 3:**
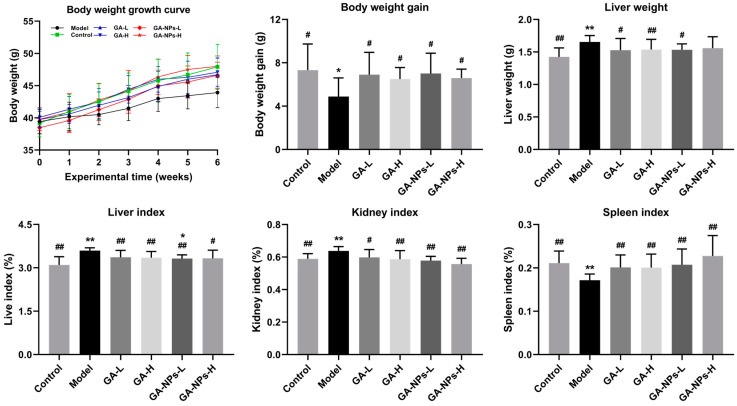
Effects of GA-NPs intervention on the body weight gain and the organ indexes in mice with excessive alcohol intake. Compared with the control group, 0.01 < ^#^ *p* < 0.05 and ^##^ *p* < 0.01. Compared with the model group, 0.01 < * *p* < 0.05 and ** *p* < 0.01.

Before gavage intervention, there was no significant difference in body weight among the experimental groups (*p* > 0.05) (Figure 3). After 6 weeks of alcohol intervention, the body weight of mice in the model group was lower than that in the other groups (*p* < 0.05), implying that alcohol exposure would affect the metabolic function of the body and inhibit the growth of mice. Compared with the control group, the liver index and kidney index of the model group were significantly increased (*p* < 0.01). In addition, it is worth noting that alcohol exposure also observably decreased the spleen index with other groups (*p* < 0.01). These results indicated that alcohol exposure may promote liver and kidney degeneration or hypertrophy and inhibit the growth and development of the spleen. Both GA and GA-NPs interventions markedly alleviated the abnormalities of organ indexes (liver, kidney and spleen indexes) caused by alcohol exposure. These results indicate that GA-NPs had a better regulatory effect than GA on the body weight and organ indexes in mice given excessive alcohol.

### 3.5. Effects of GA-NPs Intervention on Serum Biochemcial Parameters

**Figure 4:**
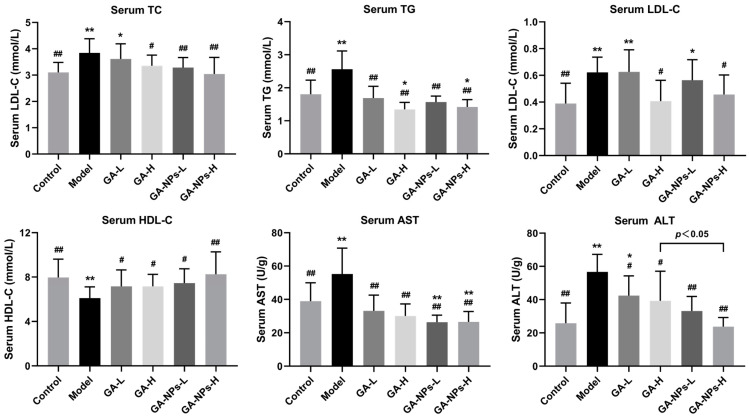
Effects of GA-NPs intervention on the serum biochemical parameters in mice with excessive alcohol intake. Compared with the control group, 0.01 < ^#^ *p* < 0.05 and ^##^ *p* < 0.01. Compared with the model group, 0.01 < * *p* < 0.05 and ** *p* < 0.01.

Compared with the control group, alcohol exposure led to abnormal elevation of serum TC, TG and LDL-C levels, as well as AST and ALT activities, but abnormal reduction of serum HDL-C level (Figure 4) (*p* < 0.01). It is generally accepted that excessive alcohol consumption can lead to fat accumulation in the liver, and abnormalities in TG metabolism. Interestingly, both GA and GA-NPs interventions decreased serum TC, TG and LDL-C levels, while significantly increasing serum HDL-C level (*p* < 0.01, versus the model group). Serum ALT activity reflects the damage of liver cell membrane to a certain extent, while AST activity indicates the damage of liver cell mitochondria. Both GA-NPs and GA treatments significantly decreased serum AST and ALT activities (*p* < 0.05, versus the model group), especially GA-NPs treatment, manifesting that the liver injury attributed to alcohol exposure could be mitigated to a certain extent. Notably, GA-NPs was more effective than GA at reducing serum ALT activities at the same dose (*p* < 0.05). Together, these results suggest that GA-NPs was effective in alleviating lipid metabolism disorders and hepatocyte damage induced by alcohol exposure.

### 3.6. Effects of GA-NPs Intervention on Liver Biochemical Parameters

**Figure 5:**
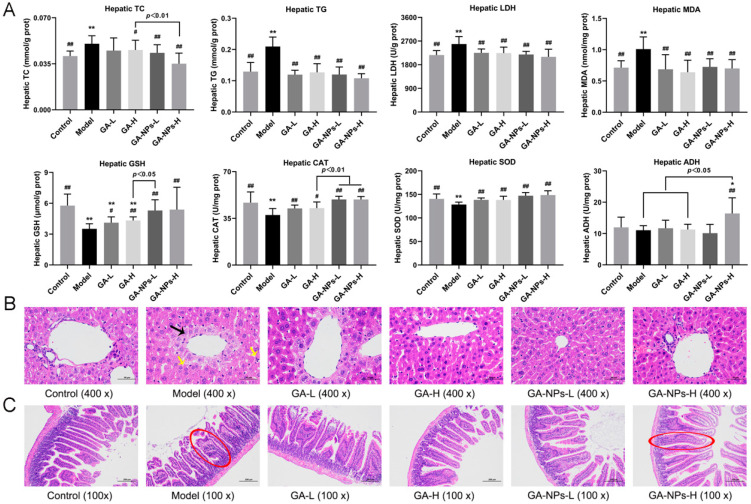
Effects of GA-NPs intervention on the liver biochemical parameters, liver and jejunum pathological morphology features in mice with excessive alcohol intake. Compared with the control group, 0.01 < ^#^ *p* < 0.05 and ^##^ *p* < 0.01. Compared with the model group, 0.01 < * *p* < 0.05 and ** *p* < 0.01. (**A**) Hepatic levels of TC, TG, GSH, CAT, MDA, SOD and LDH; (**B**) Liver pathological morphology features; black arrow: necrosis; yellow arrow: inflammation; (**C**) jejunum pathological morphology features; red outline.

The progression of ALD is closely related to the generation of highly active free radicals induced by ethanol and its metabolites. In addition, hepatic oxidative stress promotes liver injury and stimulates the inflammatory response, thus promoting the pathological progression of ALD. Previous studies have been reported that alcoholism can lead to abnormal accumulation of hepatic lipids, resulting in liver metabolic disorders and liver damage [25]. In this study, after 6 weeks of animal experiment, excessive alcohol intake induced abnormal increases in hepatic TC, TG, MDA levels and LDH activity (*p* < 0.01, versus the control group), but abnormal decreases in hepatic activities of oxidative stress-related enzymes (CAT and SOD) (*p* < 0.01) (Figure 5A), suggesting that excessive alcohol intake disrupted the lipid metabolism and stimulated the oxidative stress in the liver. Both high-dose and low-dose of GA-NPs interventions observably reduced serum TC and TG levels (*p* < 0.05, versus the model group). However, low-dose GA intervention did not significantly reduce liver TC levels in mice exposed to excessive alcohol. Abnormal increase of hepatic LDH activity is also an important indicator of liver cell damage and metabolic abnormality. Both high- and low- doses of GA-NPs significantly decreased hepatic LDH activity. The concentration of hepatic MDA reflects the degree of liver damage, and serves as a key indicator revealing the potential antioxidant capacity of the liver. Notably, both GA and GA-NPs interventions significantly reversed the abnormal activities of GSH, CAT and SOD caused by alcohol exposure (*p* < 0.05). Furthermore, the antioxidant effect of low-dose GA-NPs treatment was markedly better than that of high-dose GA treatment. GSH can effectively remove free radicals, activate a variety of metabolic enzymes, and is one of the main indicators of liver antioxidant capacity. SOD is considered to be the first line of defense against scavenging superoxide anion free radicals, producing hydrogen peroxide and oxygen. CAT promotes the breakdown of hydrogen peroxide into oxygen and water. Under the combined catalysis of SOD, CAT and GSH-Px, superoxide free radicals are decomposed into harmless water and oxygen. Therefore, the elevations of SOD, CAT and GSH-Px activities in the liver are conducive to improving the host liver health. In this study, only supplementation with high-dose GA-NPs can significantly increase liver ADH level compared to the model group. Collectedly, these findings suggest that oral GA-NPs attenuated ALI by increasing the activities of liver antioxidant enzymes, suppressing oxidative stress and improving lipid metabolism.

### 3.7. Effects of GA-NPs Intervention on Liver and Jejunum Histopathological Features

Pathological morphology analysis showed that mice without alcohol intervention (the control group) had normal hepatic lobular structure, normal hepatic cord arrangement, and no inflammatory reaction and fibrosis in hepatic parenchyma (Figure 5B). After being exposed to alcohol, the alcohol in the mouse liver was oxidized, producing acetaldehyde, which caused impairment of the hepatocyte. Compared with mice not exposed to alcohol (the control group), liver injury was evident in mice of the model group, as evidenced by necrosis of the liver tissue, infiltration of lymphocytes, neutrophils and other inflammatory cells (Figure 5B). Compared to the model group, mice treated with GA and GA-NPs exhibited a more distinct liver lobular boundary, clearer liver sinuses structure and a more orderly arrangement of liver cords. Additionally, no hepatocyte necrosis or hepatic fat accumulation was observed in GA and GA-NPs group, indicating that both GA and GA-NPs treatments were effective in improving liver injury to some extent (Figure 5B). From the above analysis of liver histopathological features, we found that GA-NPs intervention was more effective than GA in ameliorating ALI at the same dose.

Histopathological analysis of the jejunum indicated that the jejunal mucosa of non-alcohol-treated mice was clearly stratified, and the jejunal villi were neatly arranged (Figure 5C). In contrast, the jejunal villi of excessive-drinking mice were shortened, and there was disordered accumulation of neutrophils with infiltration of inflammatory cells. Compared with the mice in the model group, the intestinal damage in the mice of the GA-L, GA-H, and low-dose GA-NPs groups was improved to some extent, but the jejunal villi were still shortened and incomplete. In contrast, treatment with high-dose GA-NPs significantly increased the length of jejunal villi, which were morphologically intact, rich in cup cells and showed a significant reduction in inflammatory infiltration. The above experimental results suggest that GA-NPs have a superior protective effect on the pathological morphology of the jejunal tissues in mice given excessive alcohol compared to GA at the same dose.

### 3.8. Effects of GA-NPs Intervention on Fecal SCFAs Levels 

**Figure 6:**
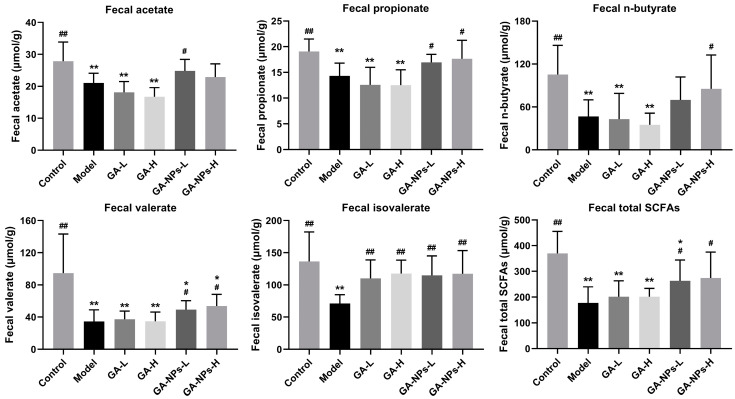
Effects of GA-NPs intervention on the fecal levels of total SCFAs (including acetic acid, propionic acid, n-butyric acid, valeric acid, and isovaleric acid) in mice with excessive alcohol intake. Compared with the control group, 0.01 < ^#^ *p* < 0.05 and ^##^ *p* < 0.01. Compared with the model group, 0.01 < * *p* < 0.05 and ** *p* < 0.01.

SCFAs, a group of fatty acids containing fewer than five carbon atoms, including acetic acids, propionic acids, n-butyric acids, valeric acids and isovaleric acids, are primarily produced through anaerobic fermentation of undigested carbohydrates. The levels of SCFAs in the intestine are largely influenced by intestinal bacterial composition and abundance. It has been reported that SCFAs are involved in the energy supply of intestinal epithelial cells, regulating the integrity of intestinal epithelial tissue and altering the permeability of the intestinal mucosal barrier [26]. During the experiment, all the experimental group mice were feasted with the same ad libitum water and common feedstuff. As shown in Figure 6, the fecal contents of acetic acid, propionic acid, n-butyric acid, valeric acid and isovaleric acid in mice of the model group were obviously decreased compared with those in the control group (*p* < 0.01). Excessive alcohol consumption can markedly reduce the concentration of SCFAs in feces, which is consistent with the results of a prior research [27]. Interestingly, both low-dose and high-dose treatments of GA-NPs significantly increased the total amount of SCFAs in feces compared to the model group (*p* < 0.05), but the difference in fecal SCFAs levels between mice treated with GA and the model group was not remarkable (*p* > 0.05). In this study, valeric acid content in the feces of mice with GA-NPs intervention was higher compared with the model group (*p* < 0.05). Valeric acid is an effective immune metabolism regulator, which is conducive to regulating intestinal barrier function and promoting intestinal health [28]. In addition, high-dose GA-NPs intervention reversed the abnormal decline in fecal n-butyric acid and propionic acid levels caused by alcohol exposure (*p* < 0.05, versus the model group). n-Butyric acid and propionic acid promote the proliferation of epithelial cells and reduce cell apoptosis, which are beneficial to maintaining the intestinal barrier function [29]. On the other hand, n-butyric acid and propionic acid perform a pivotal role in regulating lipid metabolism, helping to reduce the risk of hyperlipidemia. Notably, the fecal isovaleric acid contents in the GA and GA-NPs groups were observably higher than those in the model group. Isovaleric acid can inhibit fat production by decreasing the phosphorylation of hormone-sensitive lipase (HSL) [30]. These results suggest that oral administration of GA-NPs can effectively increase the levels of SCFAs in the feces of alcohol-exposed mice. Therefore, we hypothesized that the alleviation of ALI by GA-NPs may be partly achieved by modulating the gut microbial composition and its metabolism. 

### 3.9. Effects of GA-NPs Intervention on the Composition of Intestinal Microflora

**Figure 7:**
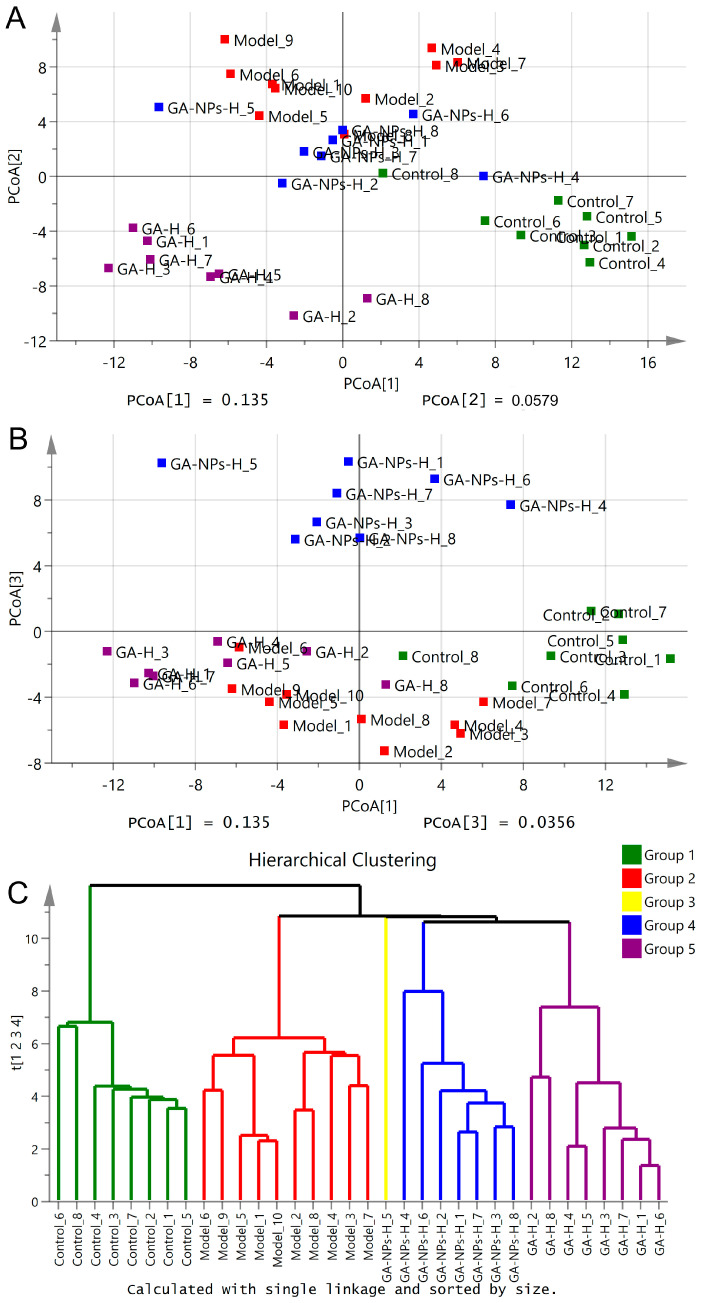
Effects of GA-NPs intervention on the composition of intestinal microbial populations in mice with excessive alcohol intake. The differences between groups were determined using a Welch’s t-test, and the Benjamini-Hochberg procedure was used to control the false-discovery rate due to multiple testing. Corrected p values are shown at right. The confidence intervals are provided to allow for critical assessment of the biological relevancy of the test results. (**A**) difference analysis of the first principal component (PCoA[1]) and the second principal component (PCoA[2]) in each group of mice; (**B**) difference analysis of the first principal component (PCoA[1]) and the third principal component (PCoA[3]) in each group of mice; (**C**) Hierarchical clustering analysis of intestinal microbiota of different experimental groups drawn based on the relative abundance at genus level.

Alcohol abuse has been reported to not only cause liver damage, but also affect the composition of intestinal microflora. Due to the “gut-liver” axis, excessive alcohol intake can cause damage to the intestinal barrier, making it easier for metabolites of harmful bacteria to be absorbed and transported to liver, further exacerbating alcohol-induced liver damage. This study employed principal coordinate analysis (PCoA) and hierarchical cluster analysis to investigate the effects of GA-NPs on the composition of intestinal microflora in mice given excessive alcohol (Figure 7). The PCoA plots exhibited that the model group were apparently separated from the control group, implying that excessive alcohol intake induced intestinal flora disorders. The GA-NPs-H group was explicitly separated from the model group along the second and third principal axis (PCoA[2] and PCoA[3]) (Figure 7A,B), showing that the composition of intestinal flora was obviously regulated by high-dose GA-NPs intervention. The hierarchical clustering plot also showed that the intestinal microbiological composition of the GA-NPs-H group was significantly different from that of the model group, suggesting that the high-dose intervention of GA-NPs indeed altered the intestinal microbiological composition of the over-drinking mice.

**Figure 8:**
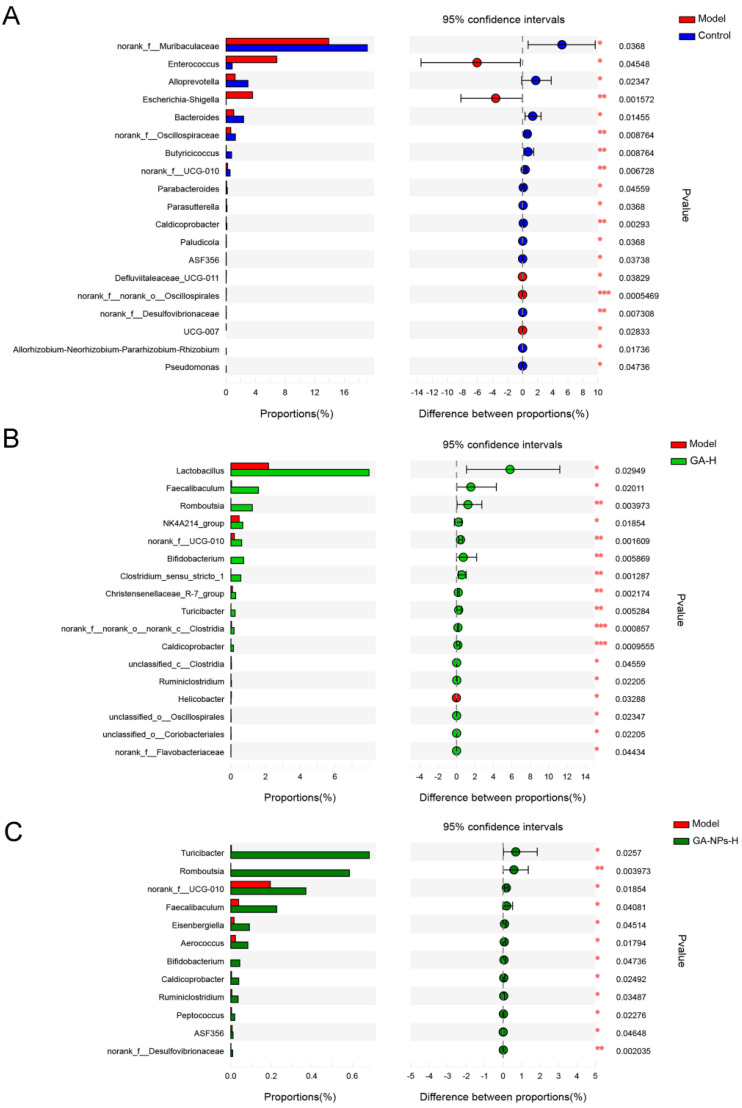
The differences of the relative abundance of microbiota between the model and control, GA-H and GA-NPs-H groups. Bars on the left represent the proportion (%) of bacterial genera and only the genus with Benjamini-Hochberg FDR corrected *p*-values indicate statistically significant differences (* *p* < 0.05). (**A**) The control group versus the model group; (**B**) The GA-H group versus the model group; (**C**) The GA-NPs-H group versus the model group.

STAMP, a software package for analyzing taxonomic profile, was used to screen key microbial phylotypes that exhibited significant differences among the control group [12], GA-H group, GA-NPs-H group and the model group at genus level (Figure 8). Compared to the control group, the model group exhibited significant changes in the relative abundance of 19 key bacterial genera. Compared with the model group, the relative abundance of 17 key bacterial genera were obviously altered by high-dose GA intervention. Especially, high-dose GA-NPs intervention distinctly altered the relative abundances of 12 key bacterial genera in mice given excessive alcohol. Similar to the GA-H group, the abundance of *Romboutsia*, *norank_f_UCG-010*, *Faecalibaculum*, *Bifidobacterium*, *Turicibacter*, *Caldicoprobacter* and *Ruminiclostridium* were significantly increased in the GA-NPs-H group compared to the model group. There is growing evidence that intestinal microbes are involved in the development of metabolic diseases such as ALI, non-alcoholic fatty liver disease (NAFLD) and insulin resistance. The intestinal microbiota plays an indelible role in regulating host metabolism by digesting complex carbohydrates and producing essential metabolites, which are the sources of energy for intestinal epithelial cells. In this work, the levels of SCFAs including n-butyric acid, valeric acid and isovaleric acid were reduced significantly after 6-week excessive alcohol intervention. It is worth noting that high-dose GA-NPs intervention significantly increased the relative abundance of the SCFAs-producing intestinal bacteria, such as *Turicibacter*, *Romboutsia*, *Faecalibaculum*, *Eisenbergiella*, *Ruminiclostridium* and *Peptococcus* in the over-drinking mice. As an important member of mammalian intestinal bacteria, the genus of *Turicibacter* was revealed to be closely associated with the host lipid metabolism. *Romboutsia* was previously reported to utilize glucose to produce beneficial metabolites such as acetic acid and isobutyric acid, which contribute to the maintenance of a healthy intestinal mucosal barrier [31]. In addition, *Romboutsia* was also found to be relation in the modulation of lipid metabolism, maintenance of intestinal epithelial barrier function and amelioration of hyperlipidemia, suggesting that *Romboutsia* may be a potential intestinal probiotic for the protection of liver-associated metabolic functions [32]. Similar to *Romboutsia*, *Eisenbergiella* was found to have the potential of degrading carbohydrates and can produce glycoside hydrolases that help to use glucose to produce SCFAs [33]. The relative abundance of *Faecalibaculum*, a predominantly butyric acid-producing bacterium in the intestine, was found significantly reduced in patients with alcoholic cirrhosis [34]. *Ruminiclostridium* and *Peptococcus* have been reported to increase the production of total SCFAs and regulate the intestinal flora structure [35]. As the main bacteria involved in lactic acid production, *Caldicoprobacter* also helps to promote the production of SCFAs in the gut [36]. *Bifidobacterium* mediates the metabolism of acetaldehyde and promotes its conversion into acetate [37]. In addition, *Bifidobacterium* and *norank_f_Desulfovibrionaceae* inhibit bile acid deposition and reduce cholesterol activity by regulating intestinal flora [38]. It was reported that *ASF356* had a significant negative correlation with serum TC, TG and LDL-C levels [28].

**Figure 9:**
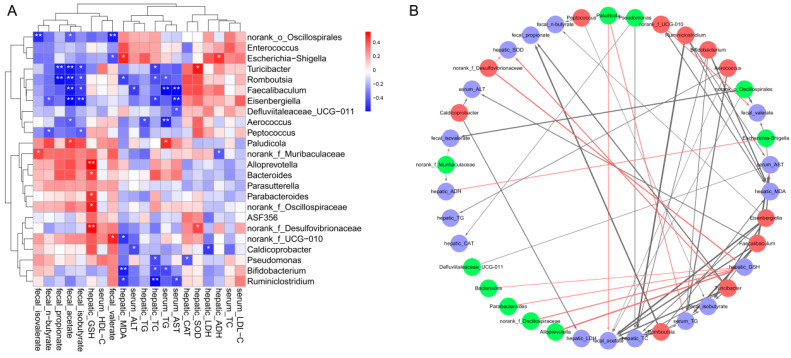
Spearman’s correlations between the key intestinal microbes and biochemical parameters. (**A**) Heatmap of correlation coefficients between the key microbes and biochemical parameters. Note: The blue square is a negative correlation, the red square is a positive correlation, ** *p* < 0.01 and * *p* < 0.05 indicate extremely significant correlation or significant correlation, respectively. (**B**) Visualization of the correlation network according to significant correlation between the biochemical parameters and the key microbes. Red nodes: the key microbes increased by high-dose GA-NPs intervention; green nodes: the key microbes reduced by high-dose GA-NPs intervention; blue nodes: the biochemical parameters. The solid red line and the black line represent positive and negative correlation, respectively. Line widths represent the strength of correlation. Only the significant edges were drawn in the network (|r| > 0.6, FDR adjusted * *p* < 0.05, ** *p* < 0.01).

The possible relationship between the key intestinal bacteria and the biochemical phenotypes was revealed by correlation heatmap and network analysis. As exhibited in Figure 9, *Turicibacter*, which prominently increased with high-dose GA-NPs intervention, had a positive correlation with hepatic SOD but a negative correlation with hepatic TC, fecal isobutyrate, fecal acetate and fecal propionate. *Romboutsia* showed a negative correlation with serum TG, hepatic TC, hepatic MDA, fecal isobutyrate, fecal acetate and fecal propionate. *Faecalibaculum* exhibited a negative correlation with serum AST and ALT, serum TG, fecal acetate and propionate. *Eisenbergiella* exhibited a negative correlation with serum AST, hepatic TC, fecal isobutyrate, fecal acetate and fecal n-butyrate. *Alloprevotella*, *Bacteroides*, *Parabacteroides*, *norank_f_Oscillospiraceae* and *norank_f_Desulfovibrionaceae* showed positive correlations with hepatic GSH. *Bifidobacterium* was found to be negatively related to serum TG, hepatic TC and hepatic MDA. However, the statistical correlation analyses still require further verification through sterile mice or bacterial intervention experiments.

### 3.10. Effects of GA-NPs Intervention on the mRNA Transcription Levels in Liver

**Figure 10:**
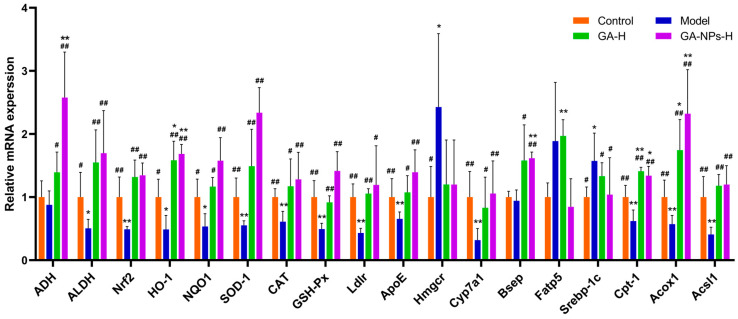
Effects of high-dose GA-NPs administration on the mRNA levels of ethanol metabolism, oxidative stress and lipid metabolism related genes in livers of mice with excessive alcohol consumption. Compared with the control group, 0.01 < ^#^ *p* < 0.05 and ^##^ *p* < 0.01. Compared with the model group, 0.01 < * *p* < 0.05 and ** *p* < 0.01.

To elucidate the protective mechanism of GA-NPs on alleviating ALI, the mRNA levels of liver genes were measured by RT-qPCR. As shown in Figure 10, high-dose GA-NPs intervention significantly up-regulated the mRNA levels of *ADH2*, *ALDH2*, *Nrf2*, *HO-1*, *NQO1*, *SOD-1*, *GSH-Px* and *CAT* genes in livers of mice exposed to alcohol (*p* < 0.01, versus model group), which are closely related to alcohol metabolism and oxidative stress. As the key organ of alcohol metabolism, liver is also the main target organ of alcohol-induced injury. Liver susceptibility to alcohol toxicity is mainly due to the high concentrations of alcohol and its oxidation product acetaldehyde in the blood [39]. The ethanol absorbed into the liver is first catalyzed by liver *ADH* to acetaldehyde, and then catalytically oxidised by *ALDH* to acetic acid, which is relatively less toxic. Both *ADH* and *ALDH* are key enzymes in the metabolism of ethanol in the body. Excessive alcohol intake inhibits *ALDH* activity and metabolic pathways responsible for converting acetaldehyde to acetic acid. This contributes to the accumulation of toxic acetaldehyde in the blood and liver, which induces the production of large amounts of ROS in hepatocytes, causing severe oxidative stress injury to the liver [40]. Interestingly, GA-NPs intervention significantly increased ethanol-induced low expressions of *ADH2* and *ALDH2*. It is worth noting that the promoting effect of GA-NPs on *ADH2* gene expression was significantly better than GA. In addition, GA-NPs treatment significantly activated the *Nrf2*/*ARE* signaling pathway and promoted the mRNA expression of antioxidant-related genes, including *Nrf2*, *HO-1*, *NQO1*, *GSH-Px*, *SOD-1* and *CAT*, in the liver. Notably, the regulation of hepatic antioxidant pathway-related genes by GA-NPs was significantly better than that by GA treatment. The above results indicate that the protective effects of GA-NPs against ethanol-induced liver injury may be partly through the activation of the *Nrf2*/*ARE* signaling pathway to promote ethanol metabolism and enhance the antioxidant capacity in the liver.

Alcohol intake can partially replace fatty acids as an energy source for the liver. It also promotes the esterification of fatty acids into TG, phospholipids and cholesterol esters, which tend to accumulate excessively in the liver. Meanwhile, acetaldehyde produced by ethanol metabolism promotes cholesterol biosynthesis by inducing endoplasmic reticulum stress, thereby leading to the development of hypercholesterolemia [41]. *Hmgcr* and *Ldlr* are two critical enzymes that control the cholesterol homeostasis in the body [42]. In this study, excessive alcohol intake significantly activated *Hmgcr* expression, thus promoting cholesterol biosynthesis in the liver. Liver is a key organ for maintaining the homeostasis of cholesterol (especially LDL-C) in the circulation, which is mainly mediated by *Ldlr*. It has been reported that alcohol-induced hypercholesterolemia is usually accompanied by abnormally decreased *Ldlr* level and elevated cholesterol level in the liver [43]. *ApoE* enables lipoprotein to enter cells for catabolism, and plays a crucial role in the transport and metabolism of cholesterol and TG. *ApoE* was also reported to bind to *Ldlr* and receptor-associated proteins to regulate plasma levels of very low density lipoprotein cholesterol (VLDL-C), HDL-C and TG [44]. In the classical pathway of bile acid synthesis, *Cyp7a1* is the rate-limiting enzyme in the conversion of liver cholesterol to bile acids, which reduces the accumulation of cholesterol, and *Bsep* is the key enzyme for cholesterol efflux. Bile salts synthesized in the liver are mainly transported to the bile ducts via *Bsep* to maintain homeostasis of cholesterol and bile acid metabolism in the liver [41]. In this study, high-dose GA-NPs intervention significantly increased the mRNA levels of *Ldlr*, *ApoE*, *Cyp7a1* and *Bsep* compared with the model group, suggesting that GA-NPs have a promising ameliorative effect on the imbalance of hepatic cholesterol and bile acid metabolism induced by excessive alcohol consumption.

In lipid metabolism, long-chain fatty acids (LCFAs) enter cells via transmembrane transport of liver-specific *Fatp5* [45]. In in vivo studies, *Fatp5* has shown compelling evidence for its role in lipid metabolism disorders and the development of fatty liver disease [46]. In the present study, GA-NPs treatment significantly inhibited the abnormally high expression of *Fatp5* in the liver after alcohol exposure, thereby inhibiting lipid transport, ameliorating the abnormal accumulation of hepatocyte lipids, and consequently decreasing the concentration of TG in the liver. *Srebp-1c* is an important regulatory factor of fatty acid synthesis. Alcohol exposure significantly up-regulates the expression of *Srebp-1c*, which in turn promotes the synthesis of fatty acid and TG in the liver. *Acsl1* is mainly responsible for the conversion of LCFAs into intracellular fatty acyl-CoA, which is further β-oxidized by *Cpt-1* in the mitochondrial membrane [47]. *Cpt-1* and *Acox1* are the key decelerating enzymes in the process of lipid β-oxidation. In this work, we discovered that alcohol exposure observably promoted the expression of liver *Srebp-1c* gene for fatty acid synthesis, and suppressed the expression of liver *Cpt-1*, *Acsl1* and *Acox1* genes for fatty acid oxidation. GA-NPs intervention significantly decreased lipid uptake and transport in liver cells and reduced lipid accumulation, which could alleviate the burden on hepatocytes to a certain extent, and restore the lipid oxidation capacity of hepatocytes, thus promoting hepatic lipid metabolism homeostasis. Moreover, the effect of GA-NPs intervention on lipid metabolism was significantly better than that of GA. In particular, the mRNA levels of *Fatp5* and *Srebp-1c* genes in liver were significantly down-regulated, and the expressions of *Acsl1* and *Acox1* genes were significantly up-regulated, suggesting that the zein-chitosan nano-delivery vehicle is beneficial for enhancing the bioactivity of GA in the body.

## 4. Conclusions

For the first time, we synthesized and characterized the co-delivery nanopolymers by using zein as the core and chitosan as the shell to deliver GA. The resulting zein-chitosan nanoparticles had a high encapsulation efficiency (92.68%) for GA. The particle size of the prepared GA-NPs was small (d = 177.20 nm) with +29.53 mV zeta potential. The protective effects and mechanisms of GA-NPs against ALI were investigated in mice given excessive alcohol, from the perspectives of biochemistry, histopathology and microbiomics combined with gene transcription analysis. The results revealed that oral administration of GA-NPs was more effective than GA in ameliorating ALI in mice exposed to alcohol. 16S amplicon sequencing demonstrated that GA-NPs was beneficial for ameliorating intestinal microbiota dysbiosis in alcohol-treated mice by increasing the proportion of *Faecalibaculum*, *Romboutsia*, *Bifidobacterium*, *Turicibacter*, *Caldicoprobacter*, *Ruminiclostridium*, etc. Correlation network analysis indicated that the key intestinal bacteria intervened by GA-NPs were closely related to the biochemical phenotypes of liver metabolism. RT-qPCR analysis indicated that GA-NPs intervention greatly reduced the extent of liver damage attributed to excessive alcohol consumption by regulating the expression of functional genes related to hepatic alcohol metabolism, oxidative stress, cholesterol and bile acid metabolism as well as lipid uptake and oxidation. Collectively, these findings revealed that GA-NPs has stronger hepatoprotective efficiency than non-encapsulated ganoderic acids. This study provides a new research idea and theoretical basis to enhance the effect of active ingredients in foods to prevent ALI. However, this study primarily utilized mice model to evaluate the therapeutic potential of GA-NPs against ALI. Extension of these findings to humans needs to be interpreted with caution due to the inherent biological differences between species, including differences in metabolism, immune responses and drug interactions. What’s more, the long-term safety of GA-NPs, including potential adverse effects and chronic toxicity, has not been extensively investigated. Future studies should include comprehensive toxicity studies and pharmacokinetic analyses to ensure the safety of long-term administration of GA-NPs in humans. Although 16S amplicon sequencing indicated beneficial effects of GA-NPs on intestinal microbiota dysbiosis in alcohol-treated mice, the specificity and long-term stability of these microbiota changes in human subjects remain uncertain. Future studies should investigate the persistence and functional implications of microbiota modulation by GA-NPs in clinical settings. In future study, the beneficial effects of GA-NPs on ALI and the mechanism of action need to be verified by clinical population trials.

## Figures and Tables

**Table 1:** Primer sequences for RT-qPCR.

Gene	Forward Primer (5′−3′)	Reverse Primer (5′−3′)
*ADH2*	AACGGTGAAKGTTCCCAAAA	ACGACCCCCAGCCTAATACA
*ALDH2*	ATCCTCGGCTACATCAAATCG	GTCTTTTACGTCCCCGAACAC
*Nrf2*	CCGGGAKCAAKCAGAKA	ACGTTGTCCCCATTTTTGCG
*HO-1*	AKCCCAGTCTATGCCCCAC	GGCGTGCAAGGGATGATTTC
*NQO1*	AACAAGCAAKCCCAGTCTATGC	AGGTAGCGGGTATATGCGTGGGCC
*SOD-1*	TTGGCCGTACAA GGTGG	CGCAATCCCAATCACTCCAC
*CAT*	TCACCCACGATATCACCAGA	AGCTGAGCCTGACTCTCCAG
*GSH-Px*	GGGACCCTGAGACTTAGAGC	AATCCGTACTAGCGCTCACA
*Ldlr*	ATGCTGGAGATAGAGTGGAGTT	CCGCCAAGATCAAAKAG
*ApoE*	AKCCGCTTCTGGGATTACCT	TCAGTGCCGTCAGTTCTTGTG
*Cyp7a1*	CCTTGGGACGTTTTCCTGCT	GCGCTCTTTGATTTAGAKG
*Bsep*	TCTGACTCAGTGATTCTTCGCA	CCCATAAACATCAGCCAGTTGT
*Fatp5*	AKTCGGGAGGCAGAAKCT	AGCGGGTCATACAAGTGAGC
*Srebp-1c*	GCCGGCGCCATGGACGAGCTGG	CAGAKGGCTTCCAGAGAGGAG
*Cpt-1*	TCCATGCATACCAAAGTGGA	TGGTAGGAGAGCAGCACCTT
*Acox*	GCCTGCTGTGTGGGTATGTCATT	GTCATGGGCGGGTGCAT
*Acsl1*	CACTTCTTGCCTCGTTCCAC	GTCGTCCCGCTCTATGACAC
Mouse 18S	AGTCCCTGCCCTTTGTACACA	CGATCCCAGGGCCTCACTA

**The full name of the abbreviations from the first column:** alcohol dehy drogenase 2 (*ADH2*), aldehyde dehydrogenase 2 (*ALDH2*), nuclear factor-erythroid 2-related factor 2 (*Nrf2*), heme oxygenase-1 (*HO-1*), quinone oxidoreductase 1 (*NQO1*), superoxide dismutase-1 (*SOD-1*), catalase (*CAT*), glutathione peroxidase (*GSH-Px*), low density lipoprotein receptor (*Ldlr*), apolipoprotein E (*ApoE*), cholesterol 7-alpha hydroxylase (*Cyp7a1*), bile salt export pump (*Bsep*), fatty acid transport protein 5 (*Fatp5*), sterol regulatory element-binding protein-1c (*Srebp-1c*), carnitine palmitoyltransferase-1 (*Cpt-1*), acyl-CoA oxidase 1 (*Acox*), acyl-coenzyme A synthetases 1 (*Acsl1*).

## Data Availability

The original contributions presented in the study are included in the article, further inquiries can be directed to the corresponding authors.
